# RNA-seq analysis unveils differential gene expression patterns following intermittent hypoxia in the mandibular condyles of growing male rats

**DOI:** 10.3389/fphys.2026.1795959

**Published:** 2026-06-10

**Authors:** Korkuan Jariyatheerawong, Jun Hosomichi, Chidsanu Changsiripun, Hideyuki Maeda, Albert Chun-Shuo Huang, Takashi Ono

**Affiliations:** 1 Department of Orthodontic Sciences, Graduate School of Medical and Dental Sciences, Institute of Science Tokyo, Tokyo, Japan; 2 Department of Orthodontics, Faculty of Dentistry, Chulalongkorn University, Bangkok, Thailand; 3 Department of Forensic Medicine, Graduate School of Medical Science, Yamagata University, Yamagata, Japan; 4 Department of Orthodontics, National Taiwan University Hospital, Taipei, Taiwan; 5 Department of Physiology, Faculty of Dentistry, Chulalongkorn University, Bangkok, Thailand

**Keywords:** cartilaginous growth, chondrocyte, infant rat, intermittent hypoxia, obstructive sleep apnea, RNA sequencing

## Abstract

**Introduction:**

Intermittent hypoxia, which is a characteristic of obstructive sleep apnea can impair mandibular growth during early development, particularly in infants. This study aimed to investigate gene expression profiles associated with mandibular cartilage growth in infant male rats exposed to IH modeling pediatric OSA.

**Methods:**

Eight-day-old male Sprague-Dawley rats were subjected to either normoxic air or IH and sacrificed after 1 week. Mandibular growth was evaluated using micro-computed tomography and histomorphometry. RNA sequencing examined differential gene expression in bone and cartilage.

**Results:**

IH caused mandibular growth deficits, including reduced total cartilage thickness in the mid- and posterior mandibular condylar regions. Comparative measurements between IH and normoxia showed thinning of all cartilage layers in these regions under IH. In contrast, the anterior region exhibited thicker proliferative and maturative layers but a thinner hypertrophic layer under IH. RNA sequencing identified 342 upregulated and 45 downregulated genes in the IH group, including key regulators of bone and cartilage metabolism, such as gremlin-2, fibroblast growth factor 2, insulin-like growth factor binding protein 2, interleukin-1B, bone gamma-carboxyglutamate protein, wingless-related integration site 2, and SRY-box transcription factor 11.

**Conclusion:**

These findings suggest that IH alters expression of critical genes in mandibular cartilage development, potentially contributing to growth deficits.

## 1 Introduction

Obstructive sleep apnea is characterized by recurrent episodes of upper airway collapse during sleep, which cause intermittent hypoxia (IH) and hypercapnia (Behrents et al., 2019). In infants, OSA-induced IH triggers harmful pro-inflammatory cascades (interleukin [IL] 1-beta [IL-1B], IL6, tumor necrosis factor [TNF]), leading to multisystem morbidity (Serebrovskaya and Xi, 2015, Wahlund et al., 2023), including cardiovascular dysregulation (Milner and Ruggins, 1989) and impaired craniofacial development (McNamara and Sullivan, 2000). Unlike sustained hypoxia (SH), where adaptive responses occur, IH is particularly detrimental (Serebrovskaya and Xi, 2015) and strongly associated with sudden infant death syndrome (Tishler et al., 1996). Moreover, infants and children have lower arousal responses to hypoxic conditions because of immature development of the respiratory system (Katz and D'Ambrosio, 2008). Growth and development of the craniofacial bone mainly occur during this period, which may have an effect on craniofacial morphology and growth in children. IH can alter the mode of breathing, which influences craniofacial growth patterns in humans and animal models at a young age (Kuma et al., 2021; Zhao et al., 2021; Kim et al., 2022) through structural changes in the craniofacial skeleton (Kuma et al., 2014) and upper airway soft tissues (Kuma et al., 2021). Regarding the effects of IH on children, it was found to retard the growth of the craniofacial bone (Oishi et al., 2016) and cartilage (Hong et al., 2021; Lekvijittada et al., 2021) and narrow the upper airway structure in young male rats (Oishi et al., 2016; Kuma et al., 2021).

Mandibular condylar cartilage is located on the articular surface of the condyle and is a fibrocartilage derived from embryonic secondary chondrocytes (Lekvijittada et al., 2021). It is essential for craniofacial development because the condyle is the center of growth of the craniofacial complex. It is vulnerable to IH during development (Mizoguchi et al., 2013). IH alters endochondral ossification, the process governing condylar growth, through chondrocyte proliferation, hypertrophy, and mineralization (Hata et al., 2017). Key regulators include extracellular matrix (ECM) components (collagen type I a1 [COL1A1], collagen type II a1 [COL2A1], matrix metalloproteinase) (Shimomura et al., 2022; Yang et al., 2023), transcription factors (SRY-box transcription factor 9 [SOX9], hypoxia-inducible factor-1α) (Lekvijittada et al., 2021; Shimomura et al., 2022), and signaling molecules (receptor activation of nuclear factor kappa-B ligand [RANKL], osteoprotegerin, transforming growth factor-β) (Hong et al., 2021; Lekvijittada et al., 2021). Although previous studies have shown that IH impairs mandibular growth in neonatal rats (Lekvijittada et al., 2021), its complete transcriptomic profile remains unexplored. Next-generation sequencing is a technology used for RNA sequencing (RNA-seq) that provides detailed gene expression data and identifies differences between physiological and pathological conditions. RNA-seq offers unparalleled resolution for identifying novel IH-responsive genes (Kukurba and Montgomery, 2015), revolutionizing skeletal biology research by revealing osteoblast differentiation pathways and emphasizing the role of epigenetic events in osteocytogenesis. In addition, RNA-seq has been applied in cartilage studies to reveal new aspects of the transcriptional regulation of cartilage growth plates. The results of many studies have shown that the disruption of epigenetic regulators can alter the expression of chondrogenic genes, such as SOX9, aggrecan (ACAN), paired-like homeodomain 1, collagen type XI,a2, and other novel transcription factors leading to the disease in cartilage tissue, and can also identify novel targets of transcription factors in chondrocytes (Ayturk, 2019).

This study aimed to clarify the gene expression profiles of impaired mandibular cartilage growth in infant male rats nurtured in IH to mimic pediatric OSA. We hypothesized that IH disrupts chondrogenic/osteogenic gene networks, leading to growth impairment.

## 2 Materials and methods

### 2.1 Experimental rats

Twenty 8-day-old male infant Sprague-Dawley rats from four 13-week-old pregnant rats represented growing rats that correlated with the neonatal stage in humans (Romijn et al., 1991); these rats were in the phase before occurrence of the peak of rapid bone growth (Luder, 1994). Because paternal age can affect offspring via the epigenetic regulation of gene expression in their pups (Yoshizaki et al., 2021), offspring from same-age mother rats were used to prevent the epigenetic regulation of skeletal growth-related genes in this study. The pups and their mothers were housed in the same plastic cages during the experimental period to reduce the stress levels of the infant rats, and they were randomly divided into two groups. The sample size was calculated using the G*power program version 3.1 based on the data from a previous study (Hong et al., 2021) of two independent groups with an α error of 0.05 and power of 0.8: the experimental group (IH group, n = 10), which experienced IH for 8 h during the 12-h “lights-on” period at a rate of 20 cycles per hour (oxygen levels fluctuating from 4% to 21% with 0% carbon dioxide) from postnatal days 8 to 15, and the normal (N) group (n = 10), which was exposed to normoxic air (Hong et al., 2021). All rats were given ad libitum access to standard chow and water and maintained under 12-h light/dark cycles at 24 ± 0.2 °C during the experimental period. Body weight and oxygen saturation were measured using a pulse oximeter (MouseOx; STARR Life Sciences Corp., Oakmont, PA, USA) placed on the neck. All pups were euthanized using isoflurane inhalation on postnatal day 15.

Ethical and animal protocols were approved by the Institutional Animal Care and Use Committee of Tokyo Medical University (approval number: R4-105, April 6, 2022) and confirmed that all experiments were performed and reported in accordance with the ARRIVE guidelines. The pups that died during the experimental period and those with technical problems in data acquisition were excluded from the analysis. To minimize bias, all data collection and analyzes were conducted by investigators blinded to the group allocation.

### 2.2 Micro-computed tomography analysis

The right side of the rat hemimandible was used for micro-computed tomography (micro-CT) and histomorphometric analyzes to evaluate mandibular condylar morphology and growth. Ten right mandibular condyles from each group were used for linear measurements and bone mineral density (BMD) analysis using an X-ray micro-CT system (micro-CT; inspeXio [SMX-100CT], Shimadzu, Kyoto, Japan) and three-dimensional image analysis software (TRI/3D-BON; RATOC System Engineering, Tokyo, Japan).

For linear measurement, resolution of 40 μm was used to scan the whole mandibles. Seven landmarks were identified on the rat mandible, and five linear distances (Kim et al., 2018; Hong et al., 2021) were calculated to evaluate mandibular growth in rat pups (**Table 1**, **Supplementary Figure 1**). Each sample was measured five times per linear distance, and the average value for each sample was calculated.

**Table 1:** Landmarks and linear measurement parameters.

Abbreviation	Definition
Landmarks on the mandible	
Co	Most superior–posterior point of the mandibular condyle
Go	Most distal point of the mandibular ramus
Mn	Most concave portion of the concavity on the inferior border of the mandibular corpus
Gn	Most inferior point on the ramus that lies on a perpendicular bisector of the line Go-Me
Me	Most inferior point of the mental protuberance
Li Mi	Most superior and anterior points on the alveolar bone of the mandibular central incisor The junction of the alveolar bone and mesial surface of the mandibular first molar
Linear distance	
Co-Li	Total mandibular length
Co-Me	Length from the condylar head to the Me
Co-Gn	Ramus height
Go-Mn	Posterior corpus length
Mi-Li	Anterior corpus length

The structure and BMD were analyzed in the cancellous bone area of the mandibular condylar head 1 mm from the condylar epiphyseal cartilage (Hong et al., 2021; Lekvijittada et al., 2021), with a resolution of 20 μm and a size of 1 × 1 × 0.2 mm (**Supplementary Figure 2**) (Lekvijittada et al., 2021) for observation of the bone microstructure of the mandibular condyles. Trabecular BMD (TMD), bone mineral content (BMC), bone volume (BV)/trabecular volume (TV), and BMC/TV were calculated using the TRI/3D-BON software (Ratoc). Each sample was measured three times, and the average value was calculated.

### 2.3 Histomorphometry of the mandibular condyles

**Figure 1:**
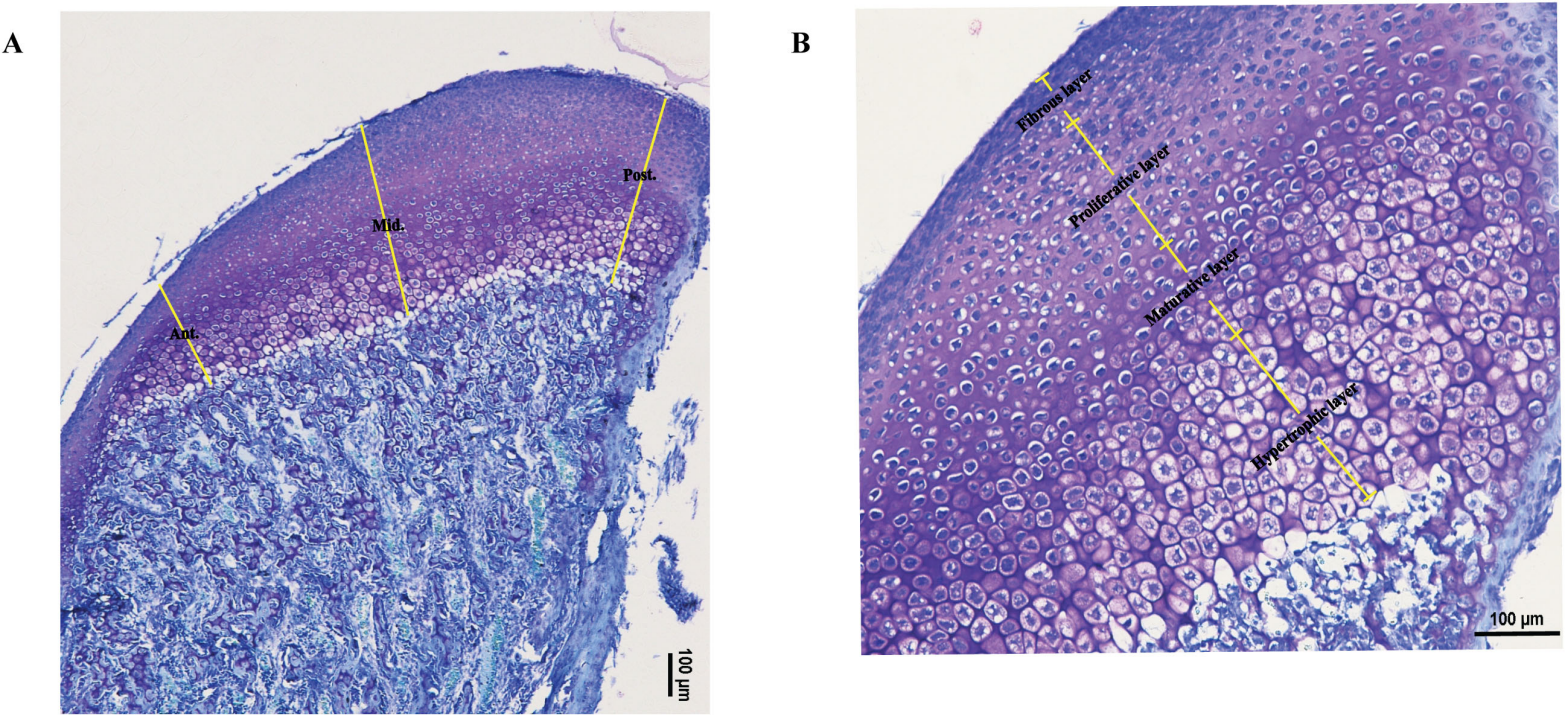
Toluidine blue staining of the mandibular condyles of male infant rats. Each sample was cut into 5 μm thickness longitudinally at the mandibular condyle area and stained with toluidine blue (pH, 4.0) for 6 min to observe condylar cartilage morphology and thickness. **(A)** Three lines were drawn through the center of the anterior, middle, and posterior portions of the mandibular condyles to measure the condylar cartilage thickness. **(B)** Mandibular condylar cartilage can be divided into four layers: fibrous, proliferative, maturative, and hypertrophic. Scale bar = 100 μm. Ant, anterior part; Mid, middle part; Post, posterior part.

The right condyles were fixed in 4% paraformaldehyde. The samples were decalcified with 10% (w/v) ethylenediaminetetraacetic acid (pH, 7.4) at 4°C for 6 weeks and embedded in paraffin. Each sample was longitudinally cut with a 5-μm thickness at mandibular condyles area. All sections were stained with toluidine blue (pH, 4.0) for 6 min to observe condylar cartilage morphology and thickness. Mandibular condylar cartilage could be divided into four layers: fibrous, proliferative, maturative, and hypertrophic (**Figure 1A**). The fibrous layer consisted of flattened fibroblast-like cells with a relatively sparse ECM. The proliferative layer was identified by flattened, densely packed chondrocytes with long axes parallel to the articular surface. The maturative layer was defined by progressive changes in cell morphology, ECM expansion, and metachromatic staining with toluidine blue. The hypertrophic layer was identified by enlarged, rounded chondrocytes with increased cytoplasmic volume being relatively 1.5-2-fold larger than maturative layer. To measure the condylar cartilage thickness, ImageJ (National Institutes of Health, USA; version 1.53k) was used in all linear distance measurements. Three lines were drawn through the center of the anterior, middle, and posterior portions of the mandibular condyles (anterior portion located on the anterior, sloped surface of the condylar head; middle portion located on the middle half of the condylar head covering the superior crest of the condyle; posterior portion located on the posterior slope of the condylar head). The total cartilage thickness and the thickness of each layer were measured (**Figure 1B**). The average of six sections per sample was then calculated.

### 2.4 RNA extraction of infant rats’ mandibular condyle and sequencing

For RNA extraction, approximately the superior one-third of the mandibular condylar head was excised. This region primarily comprises the cartilaginous layers (fibrous, proliferative, and hypertrophic zones); however, it also includes a small portion of the underlying subchondral bone. This comprehensive sampling approach was employed because the small size of the infant rat condyle makes precise isolation of cartilage alone technically challenging. Moreover, it ensured that the molecular changes within the entire condylar growth center were captured (**Supplementary Figure 3**). The mRNA library, complementary DNA (cDNA) synthesis, and sequencing were performed by Macrogen (Seoul, South Korea). Total RNA from IH and N (control) samples was pretreated with DNase to remove contaminating DNA. mRNA libraries were constructed using protocols adapted from Illumina (San Diego, CA, USA). The mRNA was purified using a TruSeq Stranded mRNA LT Sample Prep Kit (Illumina), and the isolated mRNA was randomly fragmented. RNA fragments were reverse transcribed into cDNA, and sequence adapters were ligated to both ends of the cDNA fragments. The fragments were amplified, and 200–400 bp fragments were selected. Libraries were sequenced using the NovaSeq 6000 sequencing system and NovaSeq 6000 S4 Reagent Kit (Illumina). However, two samples from the IH group and two samples from the N group were excluded because of poor RNA quality, leaving 8 samples (n = 8) per group for RNA-Seq analysis.

### 2.5 RNA-seq analysis

The raw sequencing data (fastq) were aligned to Rattus norvegicus using the RNA-Seq reference genome (Rnor_6.0) with the Alignment program HISAT2 version 2.1.0 and assembled using StringTie version 2.1.3b. After assembly, the abundance of genes/transcripts was calculated from the read count and normalized as fragments per kilobase of transcript per million mapped reads and transcripts per kilobase million for each sample. Differential gene expression was analyzed using the edgeR package (Robinson et al., 2009). Genes were considered significantly differentially expressed when the exactTest raw p-value was ≤ 0.05. Genes meeting this criterion and showing a log_2_ fold change ≤ –2 or ≥ 2 between comparison pair were selected for further analysis. A heatmap was generated to show the results of the hierarchical clustering analysis (Euclidean method, complete linkage), which clustered the similarity of genes and samples by expression levels (normalized value) from a significant list.

### 2.6 Gene ontology analysis

Differentially expressed genes were analyzed using Fisher’s exact test to determine significant enrichment in the Gene Set Enrichment Analysis within The Database for Annotation, Visualization, and Integrated Discovery version 6.7 (https://davidbioinformatics.nih.gov/) (Huang da et al., 2009a; Huang da et al., 2009b). Gene Ontology (GO) terms encompassing biological processes, cellular components, and molecular functions were considered significant at p < 0.05.

### 2.7 Protein–protein interaction analysis

The lists of biological processes related to bone and cartilage growth were selected for further examination using the STRING database (v12, https://string-db.org/) to demonstrate the protein–protein interaction (PPI) networks (Szklarczyk et al., 2023). Within the STRING network, nodes represent genes and the edges connecting these nodes represent predicted or experimentally determined PPIs. The color of each edge signifies the specific evidence supporting the interaction: light green, co-mentioned in interaction documents in public PPI databases; purple, experimentally validated PPIs (providing the most robust evidence); black, co-expressed patterns observed in expression studies (suggesting co-regulation or shared pathways); blue, interactions curated from the scientific literature (supporting potential functional connections); light blue, protein homology; and dark blue, co-occurrence across the genome.

### 2.8 Statistical analyzes

For micro-CT and histomorphometric analyzes, the data collected as the mean ± standard deviation were statistically analyzed using the IBM SPSS program. First, the normality of the data was assessed using the Kolmogorov–Smirnov test. If the distribution of the data was normal, the differences between male in the IH condition and the normal group were evaluated using an independent t-test. Otherwise, the Mann–Whitney U test was considered statistically significant for nonparametric tests when the p-value was < 0.05.

## 3 Results

### 3.1 Systemic growth and oxygen saturation measurement

Body weight and tibial length are indicators of general growth in rats. After 7 days of IH exposure, the body weight of male infant rats was significantly lower than that under normoxic conditions (**Table 2**). However, tibial length was not susceptible to IH (**Supplementary Table 1**). The O_2_ saturations of all rats were measured on day 15. The O_2_ saturation in the IH group during IH was 79.02 ± 4.68%, indicating hypoxic conditions, whereas during normoxic air, the O_2_ saturations were 98.07 ± 3.21% and 97.43 ± 1.82% in the IH and normal (N) groups, respectively, which were above 95% of the standard O_2_ saturation in normal rats (**Table 3**).

**Table 2:** Body weight changes of male infant rats in IH and normal conditions after nurturing in IH chamber and normoxic chamber for 7 days.

Body weight changes (g)		Mean diff.	p-value
IH group	N group		
3.158 ± 1.7954	9.316 ± 4.3243	–6.15789	< 0.001

IH, intermittent hypoxia; N, normal.

**Table 3:** Oxygen saturation on day 15.

	SpO_2_ (%)	
Condition	IH group (n = 10) mean ± SD	N group (n = 10) mean ± SD
Normoxic time (baseline) IH time	98.07 ± 3.21 79.02 ± 4.68	97.43 ± 1.82

IH, intermittent hypoxia; N, normal; SD, standard deviation.

### 3.2 Effects of IH on the length of mandibular condylar growth

**Figure 2:**
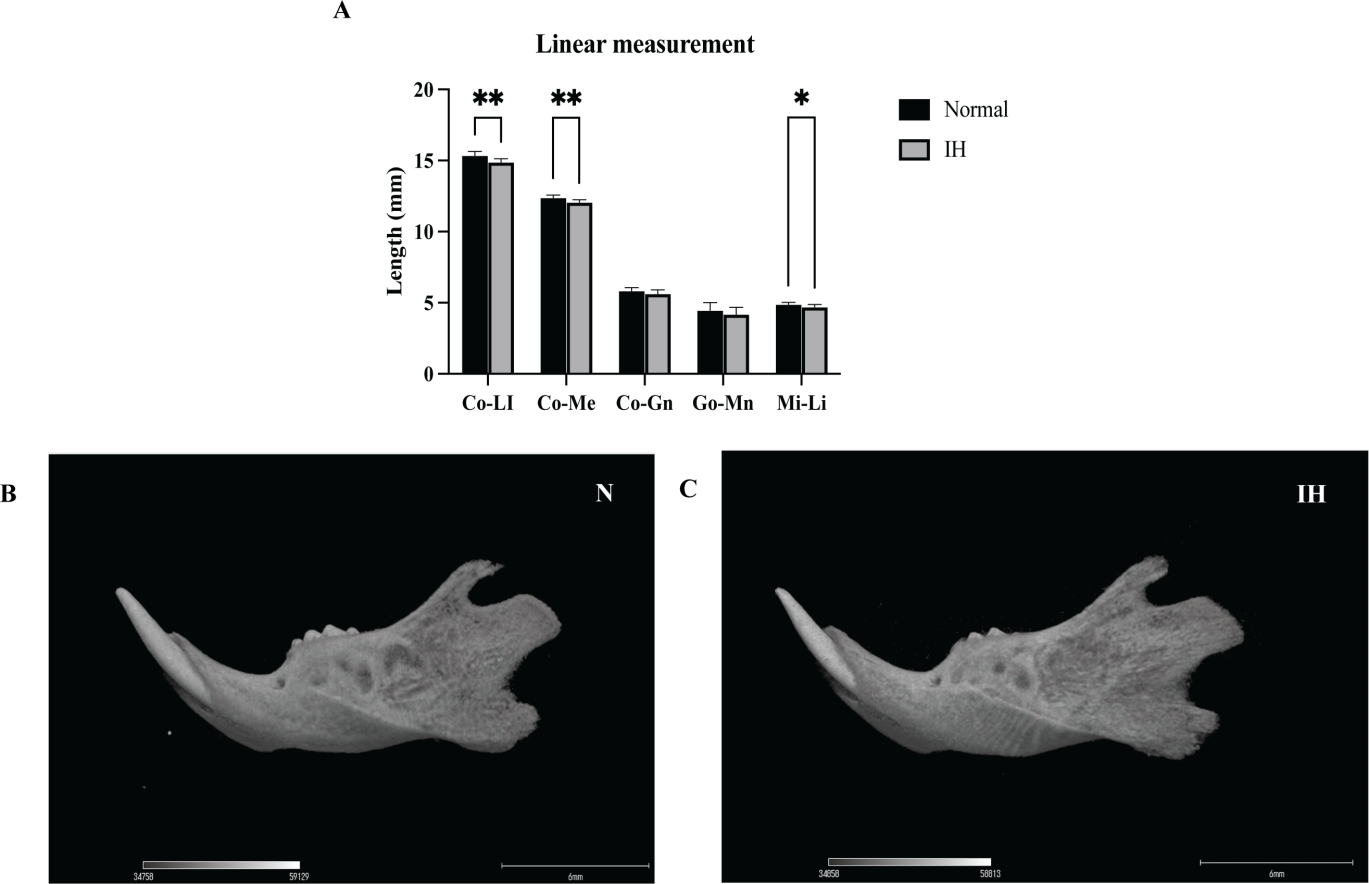
Linear measurement of mandibular length using micro-computed tomography. **(A)** All measurements were significantly lower in the IH group than in the N group for Co-Li^**^, Co-Me^**^, and Mi-Li^*^, except for Co-Gn and Go-Mn, which represent the ramus height and posterior corpus length of the mandible, respectively. Data are presented as means ± standard deviations (n = 10 per group). ^*^p < 0.05, ^**^p < 0.01 compared with control. **(B)** Three-dimensional (3D) reconstructed micro-CT images of the mandible in the control (N) group. **(C)** 3D reconstructed micro-CT images of the mandible in the IH group. The IH group displayed a visibly shortened mandibular body and altered condylar morphology compared with the control group. Scale bar = 6 mm. N, normal; IH, intermittent hypoxia.

The mandibular length was measured at five distances, as shown in **Table 1**. All measurements were significantly lower in the IH group than in the N group for every parameter (p < 0.05), except for Co-Gn and Go-Mn, which represented the ramus height and posterior corpus length of the mandible, respectively (**Figures 2A–C**).

### 3.3 Effects of IH on bone microstructural parameters of the mandibular condyle

**Figure 3:**
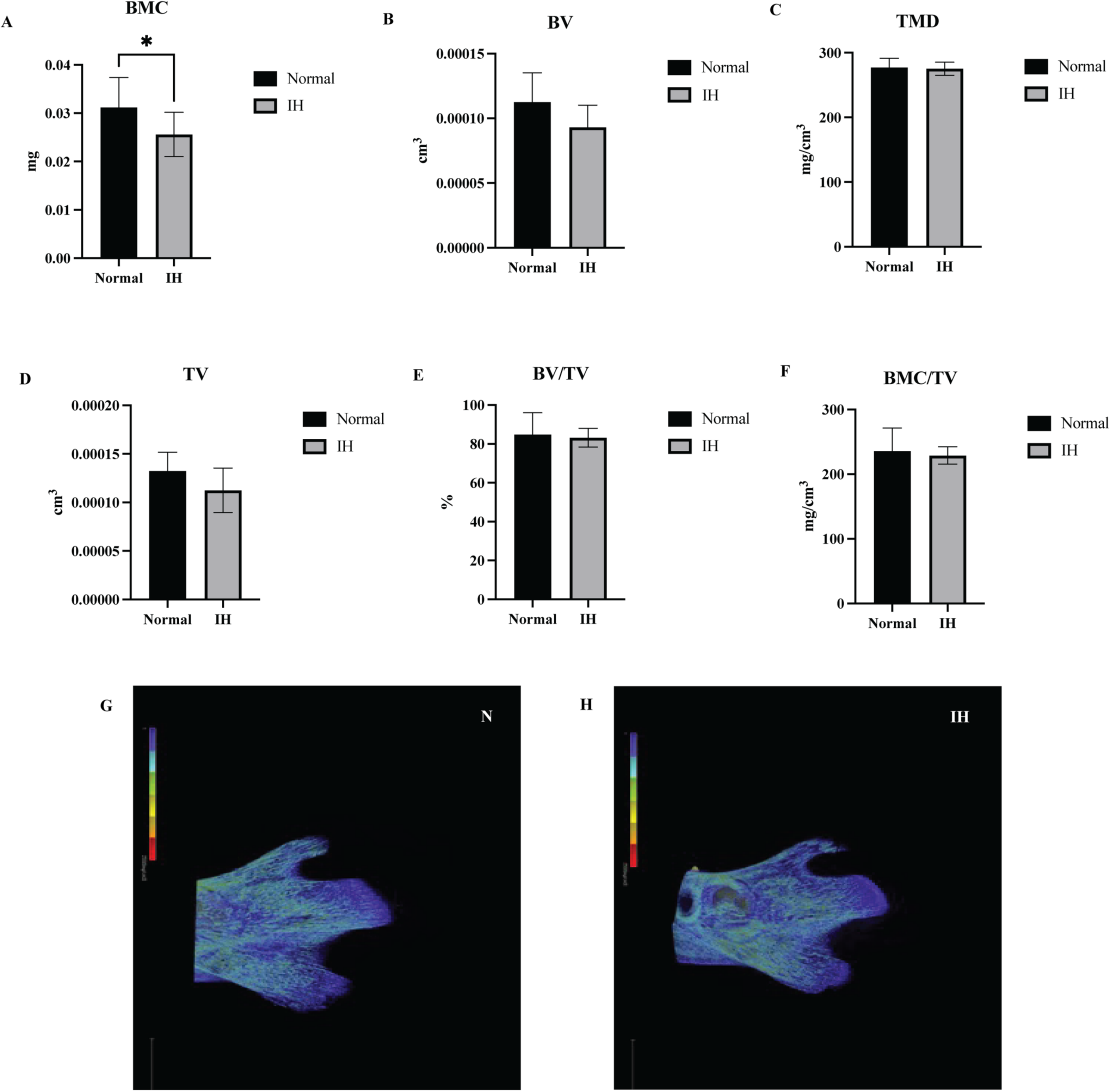
Comparative quantification of bone microstructural parameters in mandibular condyles of male infant rats in the normal and intermittent hypoxia groups. **(A)** IH significantly decreased the bone mineral content (BMC) in the mandibular condyles of male infant rats (^*^p < 0.05). Other bone microstructural parameters, such as bone volume (BV), trabecular bone mineral density (TMD), trabecular volume (TV), BV/TV, and BMC/TV, were not significantly different between the two groups. **(B)** BV is defined as the total volume of the mineralized bone tissue within the region of interest. **(C)** TMD is defined as the mineral density specifically within the trabecular bone, reflecting the degree of mineralization of the bone matrix. **(D)** TV is defined as the entire volume of the trabecular compartment, including the mineralized bone and marrow space. **(E)** BV/TV is defined as the bone volume fraction, indicating the ratio of bone volume to trabecular volume and reflecting the proportion of bone within the trabecular region. **(F)** BMC/TV is defined as a normalized measure of mineral content relative to trabecular volume. **(G)** Images represent the reconstructed trabecular bone distribution and density patterns in the N group. **(H)** Images represent the reconstructed trabecular bone distribution and density patterns in the IH group. Warmer colors (yellow to red) indicate regions of higher bone density, whereas cooler colors (blue to violet) indicate lower density. The N and IH groups exhibited low bone density in the condylar region. However, no distinct visual differences were observed between the two groups.

Bone mineralization was analyzed using micro-CT. IH significantly decreased the BMC in the mandibular condyles of male infant rats (p < 0.05). However, other bone microstructural parameters, such as trabecular bone mineral density (TMD), bone volume (BV)/trabecular volume (TV), and BMC/TV, were not significantly different between the two groups (**Figures 3A–F**). The images in **Figures 3G–H** represent the distribution and density patterns of the reconstructed trabecular bone in the N and IH groups.

### 3.4 Histomorphometry analysis on condylar cartilage growth

**Figure 4:**
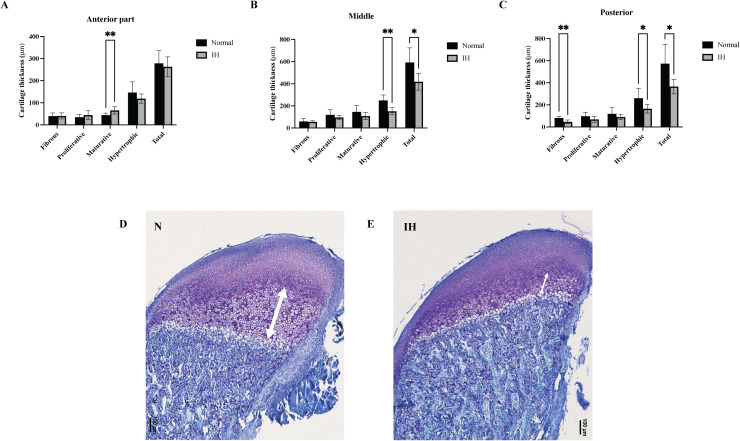
Histomorphometric analysis of the cartilaginous layer of the mandibular condylar cartilage. The figures show a comparison of the total cartilage thickness and thickness in each layer between the normal (N) and intermittent hypoxia (IH) groups [**(A)** anterior part; **(B)** middle part; **(C)** posterior part]. The total cartilage thickness was significantly reduced in the middle and posterior parts of the male rats (^*^p < 0.05, ^**^p < 0.01). However, the number of mature layers increased in the anterior part (^**^p < 0.01). **(D)** The toluidine blue-stained sections from the N group (N) **(E)** The toluidine blue-stained sections from the IH group. Histological sections demonstrate that the hypertrophic layer in the IH group is thinner than that in the N group (white arrow).

Histomorphometry was used to investigate cartilage thickness. Total cartilage thickness was significantly reduced in the middle and posterior parts of the male rats in the IH group (p < 0.05). In most layers, thickness decreased throughout the condylar cartilage, corresponding to the result of a previous study (Lekvijittada et al., 2021). However, the maturative layer in the anterior part was thicker (**Figures 4A–E**).

### 3.5 RNA-seq analysis of the temporomandibular joint of male rats

**Figure 5:**
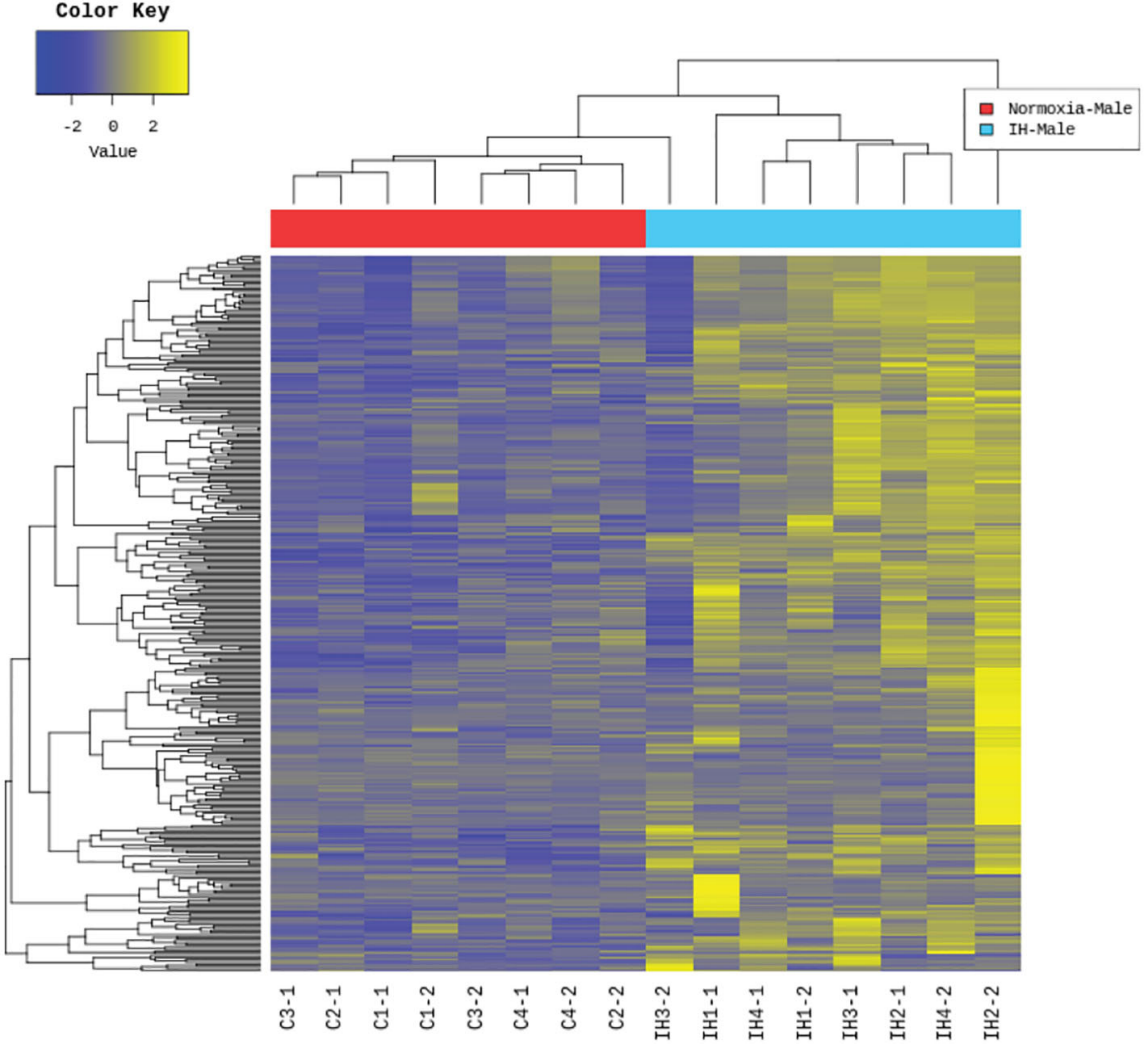
The heatmap of hierarchical clustering analysis that clustered the similarity of genes and samples based on expression level from a significant list. The yellow color indicates a high density of gene expression, whereas the blue color indicates a low density of expression. C, control/normal; IH, intermittent hypoxia.

Differential gene expression was determined by statistical analysis using fold change and an exact test for pairwise comparison. The significance result was determined by exact test raw p-value < 0.05 and ∣ fc∣ ≥ 2. The results showed 342 upregulated and 45 downregulated genes in the male IH group. The heatmap shows the results of the hierarchical clustering analysis, which clusters the similarity of genes and samples by expression levels from a significant list. The yellow color in the figure indicates a high density of gene expression, and the blue color indicates a low density of expression (**Figure 5**).

**Figure 6:**
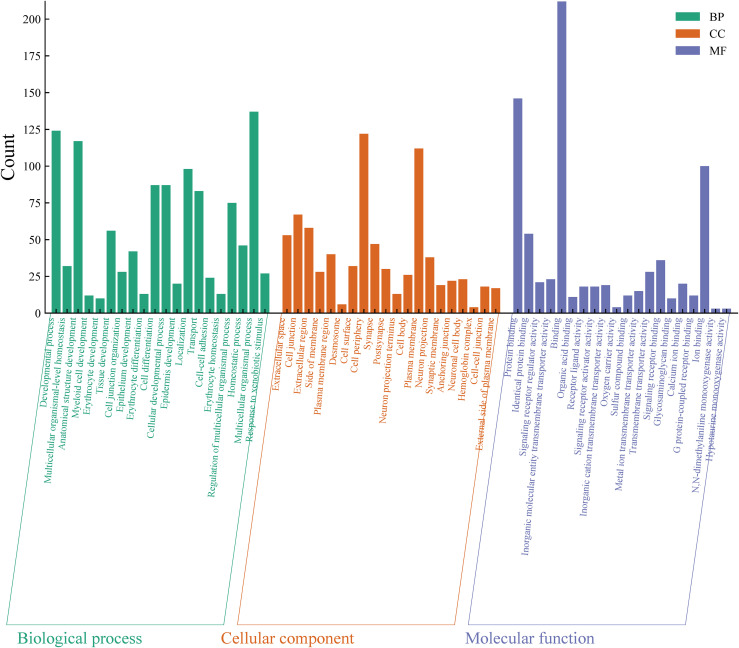
SR plot of the top 20 functional annotations of Gene Ontology (GO). All listed GO categories had an enrichment p-value of <0.05. Biological processes were the GO terms that best described the functions of the bone and cartilage-related genes in our study. Developmental processes are the most important processes related to bone and cartilage growth. Biological processes, cellular components, and molecular functions are shown in green, orange, and purple, respectively.

From a list of >300 genes, the program divided significant genes into three categories according to GO functional analysis: biological processes, cellular components, and molecular functions. The SR plot shows the list of 20 functional annotations of GO of biological processes, cellular components, and molecular functions according to the p-value (p < 0.05) (**Figure 6**). Biological processes were the GO terms that best described the functions of the bone and cartilage-related genes in our study. Developmental processes are the most important processes related to bone and cartilage growth. In this category, 124 genes are expressed, including gremlin-2 (Grem2), fibroblast growth factor 2 (FGF2), insulin-like growth factor binding protein 2 (Igfbp2), IL-1B, bone gamma-carboxyglutamate protein (BGLAP), wingless-related integration site 2 (Wnt2), and SRY-box transcription factor 11 (SOX11), which are essential for the rat’s mandibular growth regulation. The relationships between these genes are shown in the Search Tool for the Retrieval of Interacting Genes (STRING) network (https://string-db.org/) (**Supplementary Figures 4A, B**) (Szklarczyk et al., 2023). FGF2, Igfbp2, IL-1B, BGLAP, Wnt2, and SOX11 genes were co-expressed with each other, with co-expression score of 0.058–0.075. The highest score was the interaction between IL-1B and FGF2, which was 0.075 (**Supplementary Figure 5**).

**Figure 7:**
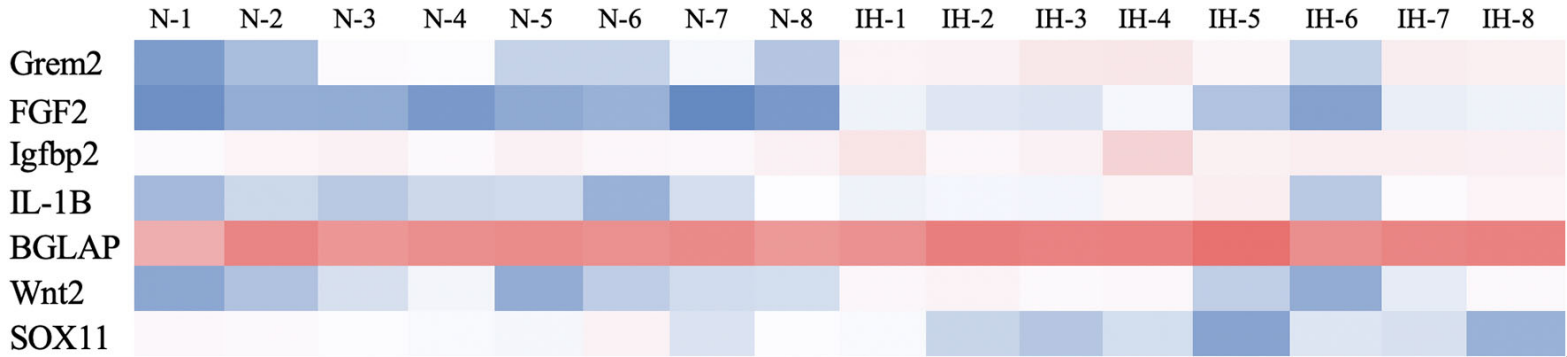
The heatmap of the bone- and cartilage-related gene expression in developmental process, including fibroblast growth factor 2, insulin-like growth factor binding protein 2, interleukin-1B, bone gamma-carboxyglutamate protein, wingless-related integration site 2, and SRY-box transcription factor 11. BGLAP was highly expressed in both groups and was upregulated in the intermittent hypoxia (IH) group. Grem2, FGF2, Igfbp2, IL-1B, and Wnt2 were upregulated (pink or light blue color), but SOX11 was downregulated (blue color) in the IH group.

Grem2 indirectly interacted with other genes via the bone morphogenetic protein (BMP) family (**Supplementary Figure 4B**); however, BMP displayed no significant changes in our study. From the heatmap, BGLAP was highly expressed in both groups but was significantly upregulated in the IH group. Grem2, FGF2, Igfbp2, IL-1B, and Wnt2 were upregulated, but SOX11 was downregulated (**Figure 7**).

## 4 Discussion

IH is widely recognized for its adverse effects on skeletal development, particularly on mandibular growth in male growing rats (Hong et al., 2021; Lekvijittada et al., 2021) by inducing premature chondrocytic hypertrophy (Meretoja et al., 2013) and disrupting the normal process of endochondral ossification, which results in significantly shortened mandibular length. Consistent with this, our study confirmed the hypothesis that IH indirectly affects mandibular growth by specifically reducing the thickness of hypertrophic chondrocyte layers, especially in the middle and posterior regions. Notably, the anterior portion of the condyle exhibited an increase in the proliferative and mature cell layers, suggesting that IH may have delayed the maturation of cartilage in the anterior segment during a crucial period of craniofacial development. Consequently, the morphology of the mandible was altered in the condylar–ramus region, leading to decreased total mandibular length (Co-Li), length from the condylar head to the menton (Co-Me), and anterior corpus length (Mi-Li). However, ramus height (Co-Gn) and posterior corpus length (Go-Mn), which are areas related to masticatory muscle development, were not affected (Hichijo et al., 2014), indicating that IH primarily influences cartilage-related bone growth rather than muscle-driven morphological changes.

While the significant weight loss in the IH group suggests a systemic metabolic impact (Schwartz et al., 1998; Nieminen et al., 2002; Balbani et al., 2005), we found that tibial growth was not significantly susceptible to IH. This finding indicates that the observed mandibular growth impairment is not merely a secondary effect of generalized growth retardation. Preservation of the tibial length, which is governed by primary cartilage, contrasts with significant retardation of the mandibular condyle. This difference likely stems from the unique characteristics of secondary cartilage, which make the mandibular growth center specifically vulnerable to hypoxia-induced dysregulation (Robinson et al., 2015).

Two-week-old male rats, which were at a stage equivalent to the weaning period in rodents, were selected to minimize the influence of solid food mastication and masticatory muscle activity on mandibular growth. At this age, rats begin transitioning from mother’s milk to solid food. This period was chosen to minimize the impact of chewing (Ghasemi et al., 2021). Thus, mandibular growth during this stage was attributed to genetic and environmental factors rather than functional factors. Micro-CT analysis provided further insights into the effects of IH on bone mineralization in the mandibular condyle. Previous studies on juvenile and adolescent rats have suggested that IH has a greater effect on bone metabolism during later periods of bone growth than during younger stages (Hong et al., 2021). This study showed no significant changes in trabecular bone density and other bone microstructural parameters, except for BMC. However, some authors have reported a positive effect of IH on BMD (Oishi et al., 2016; Lekvijittada et al., 2021), a finding that remains controversial.

The condylar cartilage serves as the growth center of the mandible, and chondrocyte development within its cartilaginous layer influences mandibular condylar growth. A previous study reported that although hypertrophic chondrocytes were decreased in cell layers, compared with those in the normal group, their proportions were increased (Lekvijittada et al., 2021). This study also found a significant reduction in hypertrophic layers, which are the thickest part of the condylar cartilaginous layer, leading to an overall reduction in cartilage thickness. At the molecular level, this phenomenon can be attributed to the upregulation or downregulation of specific genes identified in our study.

FGF2, which was significantly differentially expressed in our study, plays an important role in bone and cartilage metabolism. Upregulation of FGF2 inhibits chondrogenesis by reducing the proliferation and differentiation of chondrocytes in the mandibular condyles (Ogawa et al., 2003). Furthermore, FGF2 promotes rat osteoblast differentiation together with vascular endothelial growth factor and bone morphogenetic protein (BMP)-2, potentially leading to premature endochondral ossification, although the BMP-2 gene itself did not show significant differences in our RNA-Seq data. Grem2 functions as a critical modulator by limiting BMP signaling (Zuniga et al., 2011) to prevent excessive differentiation (Gazzerro and Canalis, 2006). In the present study, IH-induced upregulation of Grem2 may have resulted in functional suppression of BMP signaling, thereby delaying chondrocyte progression from the proliferative stage to terminal hypertrophic differentiation. This accumulation of undifferentiated cells manifested as the increased thickness of the anterior cartilage layers. In contrast, the middle and posterior regions, which are more vulnerable to IH, exhibited overall thinning, dominating the overall growth retardation in the mandible. Consistent with this regulatory role, Grem2 suppression has been shown to increase BMP-2-induced osteogenesis in human bone marrow stem cells and animal models (Meretoja et al., 2013; Nilsson et al., 2024). Conversely, Grem2 overexpression has the opposite effect in mouse femurs (Wang et al., 2017), leading to decreased bone density. Grem2 also inhibits mesenchymal stem cell (MSC) differentiation into chondrocytes under hypoxic conditions and reduces proteoglycans and the expression of chondrogenic differentiation markers, such as COL1A1, COL2A1, SOX9, and ACAN (Zhou et al., 2021), contributing to the decrease in chondrocytic cell layers in the IH group.

While FGF2 can promote premature endochondral ossification in certain contexts (Novais et al., 2021), its upregulation under IH—coupled with the significant increase in Grem2—likely reflects a dysregulated growth environment that inhibits chondrocyte maturation (De Luca and Baron, 1999). The region-specific histological changes—thinning in the middle and posterior portions versus thickening in the anterior portion—highlight the complex response of the condyle to IH. The increased thickness in the anterior region likely resulted from the accumulation of immature chondrocytes caused by a terminal differentiation delay rather than accelerated growth. This interpretation was supported by the high expression of Grem2, which limits BMP-mediated maturation. Consequently, failure of these cells to progress through the endochondral pathway ultimately contributes to the overall reduction in mandibular dimensions.

Our RNA-Seq analysis further revealed the upregulation of Igfbp messenger RNA (mRNA). It is expressed in the outer fibrous cell layer of the developing mandibular condyle (Yokota et al., 2008). A previous study on long bone growth indicated that Igfbp2 could mediate proliferation and matrix synthesis and reduce hypertrophic maturing chondrocytes (Shibata et al., 2012). The upregulation of this gene in the present study further emphasizes the effect of Igfbp2 on mandibular condylar growth.

In terms of inflammatory signaling, increased expression of IL-1B along with TNF-α acts in synergy to locally suppress longitudinal growth, decrease chondrocyte proliferation, and increase apoptosis in the long bone (Mårtensson et al., 2004). These cytokines may act synergistically in IH-exposed environments, promoting unfavorable skeletal outcomes. Additionally, our findings revealed the upregulation of BGLAP (osteocalcin), which plays a dual role in bone metabolism and can interact with Msh homeobox 2 during osteoblast differentiation (Kaneto et al., 2016). Its carboxylated form is one of the main organic components of the bone matrix and acts as a negative regulator of bone formation, limiting bone formation without impairing bone mineralization (Hassan et al., 2004). The upregulation of BGLAP under IH suggests increased bone turnover activity (Menanteau et al., 1982; Neve et al., 2013). However, the significant reduction in BMC, despite unchanged BMD, points toward the decoupling of bone formation and mineralization. This state may represent a form of high-turnover mineralization failure, where IH-induced stress accelerates metabolic activity but impairs effective deposition of the bone matrix. This imbalance likely contributed to the restrained mandibular growth observed in the IH group. The Wnt/β–catenin pathway promotes the differentiation of osteoblast-like cells by upregulating the expression of RANKL (Nie et al., 2012), which is increased at the mRNA level in rats exposed to IH (Hong et al., 2021).

However, our RNA-Seq results showed the downregulation of SOX11 expression. The SOX family proteins play essential roles in biological processes and organogenesis. The SOX11 deletion mouse model exhibits developmental defects in the craniofacial skeleton resulting from decreased bone formation and osteogenesis (Gadi et al., 2013). SOX11 also promotes chondrogenesis by regulating β-catenin, activating BMP/Smad signaling in MSCs, and activating RUNX2, which promote MSC migration (Xu et al., 2019). Notably, the SOX11 downregulation observed in our study was correlated with mandibular condylar growth retardation and decreased chondrogenic activity in rats exposed to IH.

Although this study utilized bulk RNA-seq of the entire mandibular condyle, yielding a global transcriptional output, the findings histologically manifested as region-specific outcomes. The overall molecular profile, characterized by the downregulation of pro-chondrogenic factors like SOX11, aligned with the significant thinning observed in the middle and posterior regions. These areas comprise the bulk of the condylar cartilage and are primary drivers of mandibular lengthening. In contrast, thickening of the anterior cartilage layers suggests a localized differentiation arrest that, while histologically evident, does not counteract the dominant growth retardation signals identified at the transcriptomic level. This dissociation emphasizes that the global transcriptional deficit under IH conditions is the primary determinant of overall mandibular growth impairment.

This study has some limitations. First, the nocturnal nature of rodents, whose sleep–wake patterns are opposite to those of humans, implies that they are active at night and sleep during the daytime (Hoshino et al., 2016). Our study simulated IH exposure in alignment with inherent circadian rhythms. These differences may influence the generalizability of our findings. Second, this study involved only male rats to maintain consistency with the established neonatal IH model (Lekvijittada et al., 2021). We acknowledge that the influence of sex hormones is likely minimal at the early postnatal stage examined in this study, as the onset of the estrous cycle in rats typically occurs several weeks later (Mabry et al., 2024). Therefore, the use of male rats was not intended to control for hormonal variability but instead reflects methodological consistency with the established model and practical constraints related to specimen availability. Future studies including both sexes are needed to evaluate potential sex-specific responses on IH condition during early postnatal development. Third, protein-level validation using tools such as immunohistochemistry for the differentially expressed genes identified by RNA-seq was not performed. In addition, the extremely small size of the mandibular condyles in early postnatal rats limited the availability of high-quality serial sections, thereby constraining the feasibility of performing additional protein-level analyzes. While our transcriptomic data provides a comprehensive overview of the molecular pathways affected by IH, further studies are necessary to confirm whether these gene expression changes translate into corresponding protein-level and functional alterations. Nevertheless, the observed transcriptomic changes in key regulatory markers are highly consistent with the findings of previous reports. For instance, downregulation of SOX11 and COL2A1 aligned with the histomorphometric thinning observed in our IH model, a correlation previously validated at both the mRNA and protein levels in secondary cartilage stress models (Kan et al., 2013; Lekvijittada et al., 2021). Furthermore, localized upregulation of Grem2 as a BMP antagonist provides a biologically plausible explanation for the observed maturation arrest, consistent with its established role in skeletal patterning (Gazzerro and Canalis, 2006; Zuniga et al., 2011). Therefore, our RNA-seq findings likely reflect the actual pathophysiological state of the condylar tissue. Moreover, the differential expression of key regulators, such as FGF2 and SOX11, was interpreted in the context of their established roles in cartilage biology. The downregulation of SOX11 is consistent with reduced chondrogenic activity, whereas the upregulation of FGF2, which is known to suppress chondrocyte proliferation and maturation in condylar cartilage, further supports impaired cartilage growth under IH. Together, these findings demonstrate a close correspondence between transcriptional changes and the observed histological phenotype.

Although protein-level validation was not performed in the present study, bulk RNA-seq provides a robust and quantitative assessment of global transcriptional activity (Wang et al., 2009). While post-transcriptional regulation may influence protein abundance, mRNA expression profiles remain widely accepted as reliable indicators of pathway activation and cellular state, particularly when interpreted in conjunction with phenotypic data. Indeed, although mRNA and protein levels are not always perfectly correlated (Maier et al., 2009; Gedeon and Bokes, 2012), numerous studies have demonstrated that transcriptional alterations frequently parallel functional changes at the tissue level, especially in developmental and stress response contexts (Schipani et al., 2001; Pfander et al., 2005).

Furthermore, IH was applied for 7 consecutive days in 2-week-old infant rats, whose developmental stage roughly corresponds to early infancy in humans. This short exposure period significantly retarded mandibular growth in a previous study (Lekvijittada et al., 2021). However, the long-term effects of early-life IH on mandibular development should be explored in the future to assess bone remodeling and functional adaptation over time. Moreover, the etiology of OSA in humans is multifactorial and influenced by anatomical, neurological, and behavioral factors beyond IH alone. Consequently, the direct translation of these findings to human conditions should be interpreted with caution. Mechanistically, further investigation should explore the dynamic roles of FGF2, Grem2, Wnt/β-catenin, and SOX11 in greater detail. Investigating these pathways using functional assays may clarify whether the observed gene expression patterns translate into permanent skeletal remodeling or are part of a transient adaptation to IH during development. Time-course and pathway-targeted studies may reveal whether these molecular alterations lead to permanent skeletal remodeling.

From a translational perspective, the molecular alterations identified in this study suggest potential targets for mitigating IH-induced impairment of mandibular growth. In particular, the upregulation of Grem2 and downregulation of SOX11 highlight a shift toward impaired chondrocyte maturation. Therefore, modulation of these pathways may represent a promising therapeutic strategy. Specifically, suppressing Grem2 activity to restore BMP signaling or enhancing SOX11 expression to support chondroprogenitor function and differentiation could promote normal cartilage development under IH conditions. While such approaches remain speculative, advances in localized drug delivery and gene modulation techniques raise the possibility of enabling site-specific targeting of the mandibular condyle while minimizing systemic effects. Future studies are warranted to investigate these strategies *in vivo* and to determine whether targeted modulation of these pathways can rescue the observed growth deficits induced by IH.

Taken together, IH impairs mandibular growth in male infant rats by reducing mandibular length, including the condyle–ramus region, and decreasing the thickness of the mandibular condylar cartilaginous layer, notably affecting hypertrophic chondrocytes. This phenomenon is elucidated by gene expression analysis using RNA-Seq. Our findings provide, for the first time, a comprehensive genetic profile of the mandibular condylar cartilage in growing male rats exposed to intermittent hypoxic conditions. Key genes involved in bone and cartilage metabolism during developmental processes, such as Grem2, FGF2, Igfbp2, IL-1B, BGLAP, Wnt2, and SOX11, were differentially expressed, contributing to mandibular cartilaginous growth deficit observed under IH conditions.

## Data Availability

The original contributions presented in the study are publicly available. This data can be found here: https://www.ncbi.nlm.nih.gov/geo/query/acc.cgi?acc=GSE333533.
