# Supplementary material for: RNA-seq analysis unveils differential gene expression patterns following intermittent hypoxia in the mandibular condyles of growing male rats
Source: Front Physiol. 2026 Jun 10;17:1795959. doi: 10.3389/fphys.2026.1795959 (PMC13292768; doi:10.3389/fphys.2026.1795959)
Supplement: Supplementary file 1 [file DataSheet1.docx]

Supplementary Material

RNA-Seq analysis unveils differential gene expression patterns following intermittent hypoxia in the mandibular condyles of growing male rats

Korkuan Jariyatheerawong^1,2^, Jun Hosomichi^1*^, Chidsanu Changsiripun^2^, Hideyuki Maeda^3^, Albert Chun-Shuo Huang^4^, Takashi Ono^1,5^

*** Correspondence:**Jun Hosomichi
hosomichi.orts@tmd.ac.jp


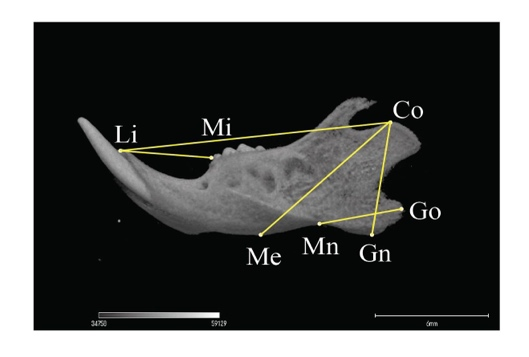


**Supplementary Figure S1.** Cephalometric landmark and linear distance on the mandibles. For definition, refer to Table 4. Scale bar = 6 mm.


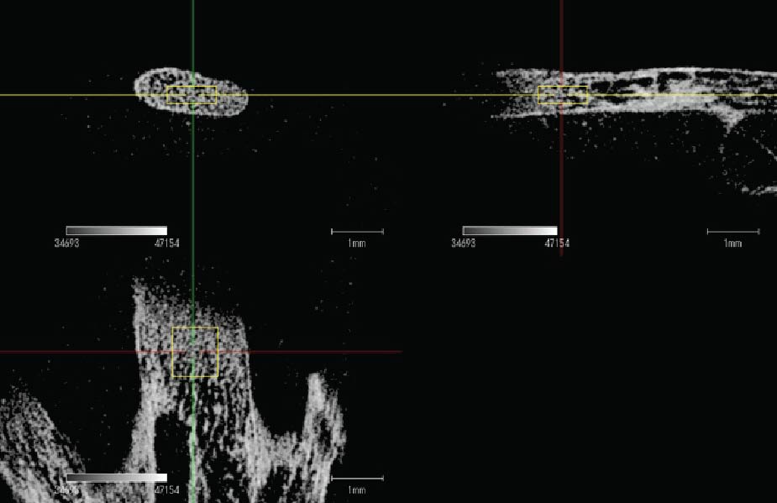


**Supplementary Figure S2.** Micro-computed tomography analysis landmarks. Bone mineral density analysis of the cancellous bone of the mandibular condylar head 1 mm from the condylar epiphyseal cartilage with a resolution of 20 μm and size of 1 × 1 × 0.2 mm (yellow box). Scale bar = 1 mm.


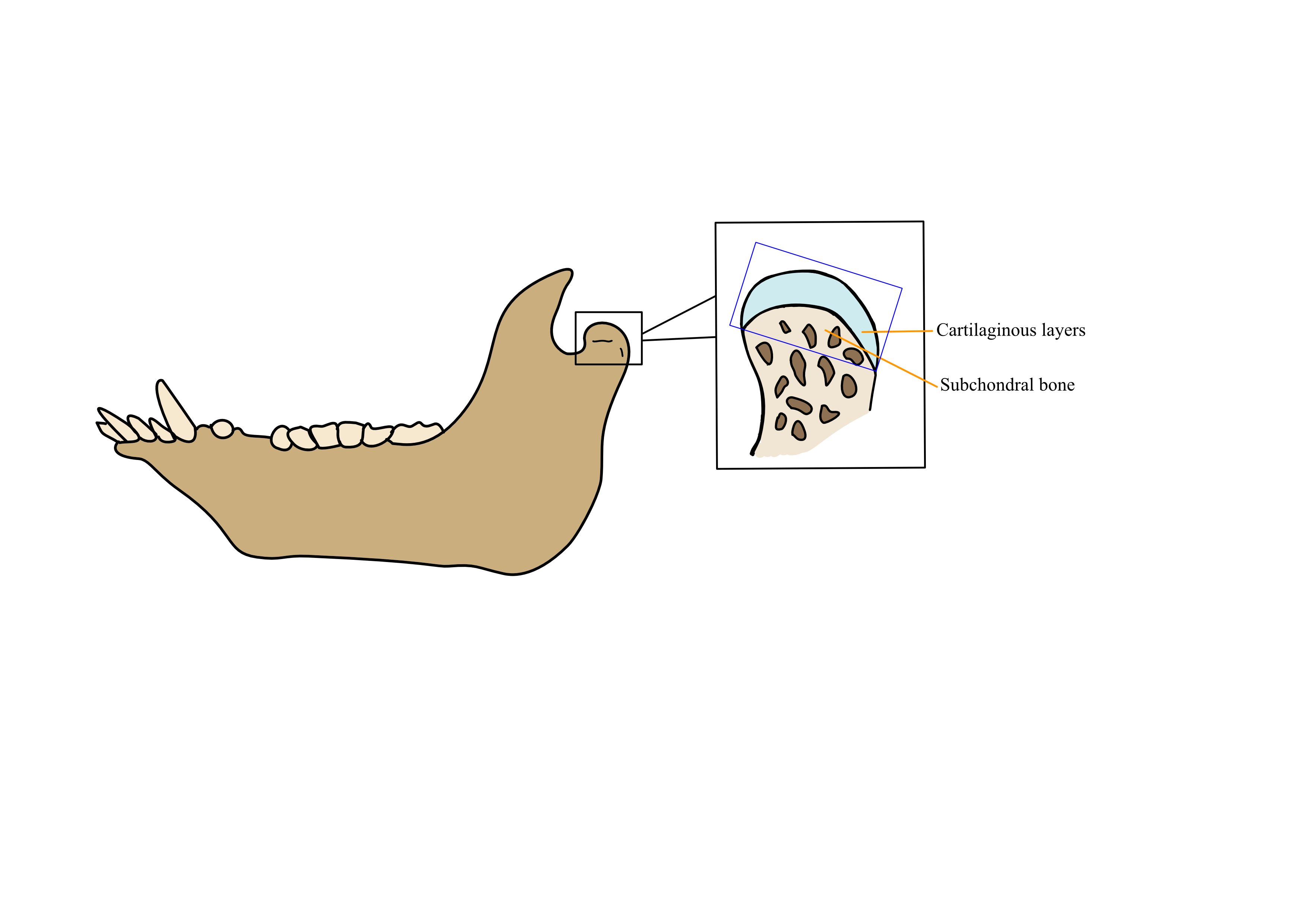


**Supplementary Figure S3.** The diagram demonstrated the superior one-third of condylar head which was collected from left mandibular condyle for RNA-Seq analysis. This region primarily consists of the cartilaginous layers (fibrous, proliferative, and hypertrophic zones) and a small portion of underlying subchondral bone (blue box).


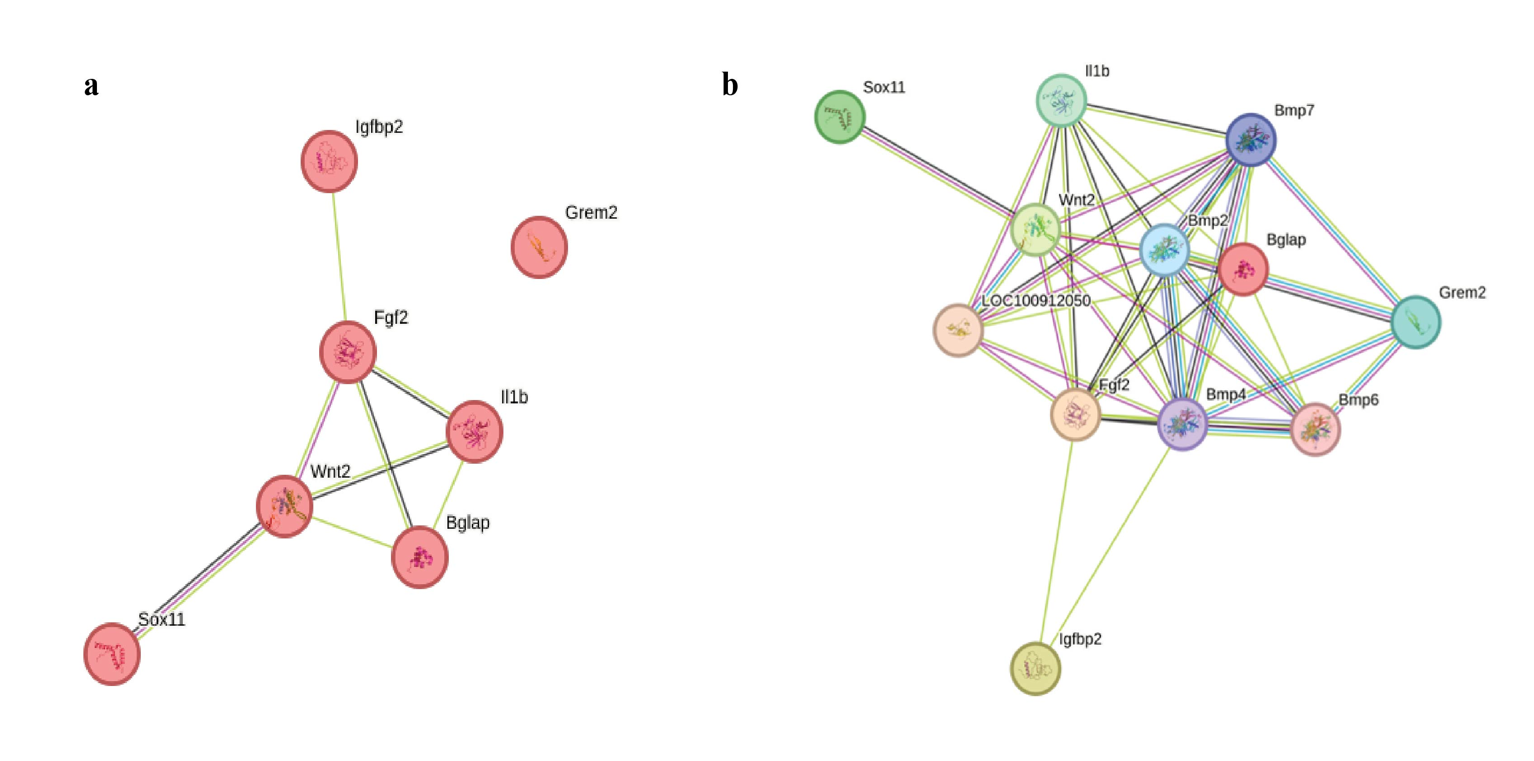


**B**

**A**

**Supplementary Figure S4.** Network analysis of the significant genes selected from the developmental process functional group generated using STRING database (v12, https://string-db.org/). **(A)** All selected genes were associated and co-expressed with each other, except for gremlin-2 (Grem2). **(B)** Grem2 indirectly interacts with other genes via the BMP family.


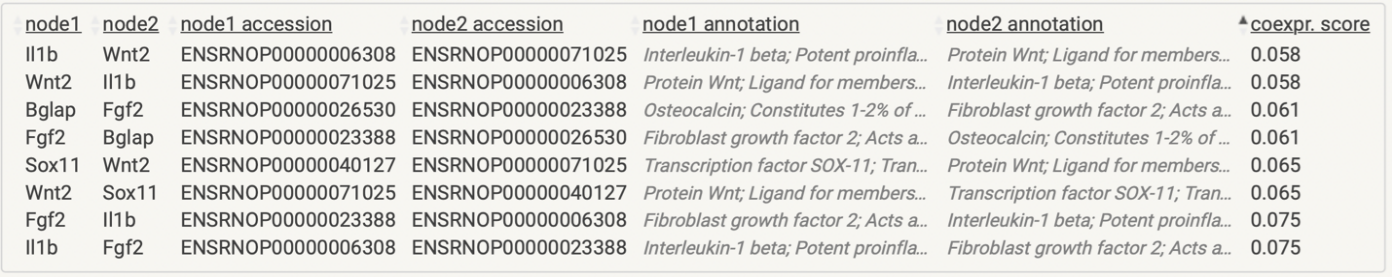


**Supplementary Figure S5.** Co-expression analysis of fibroblast growth factor 2 (FGF2), insulin-like growth factor binding protein 2 (Igfbp2), interleukin (IL)-1B, bone gamma-carboxyglutamate protein (BGLAP), wingless-related integration site 2 (Wnt2), and SRY-box transcription factor 11 (SOX11) genes (https://string-db.org/).

| Tibial length (mm) | | Mean diff. | p-value |
| --- | --- | --- | --- |
| IH group | N group |  |  |
| 12.4728 ± 0.33296 | 12.7712 ± 0.40232 | –0.29838 | 0.095 |

**Supplementary Table S1.** Tibial length of male infant rats in IH and normal conditions at day 15. IH, intermittent hypoxia; N, normal.
